# Dr. John H. Pickett

**Published:** 1898-09

**Authors:** 


Dr. John H. Pickett, of Elizabeth, N. J., died at his residence in that city August 9, 1898, aged 68 years. He was a native of Buffalo and received his medical degree from the Buffalo University Medical College in 1860. When the civil war came he enlisted as hospital steward in the 49th Regiment New York Volunteer Infantry. He was promoted to be assistant-surgeon, December 16, 1862, and was mustered out with the regiment October 18, 1864. The legislature of New York awarded a medal to Dr. Pickett for noteworthy services at Gettysburg. Soon after the war closed he went to Elizabeth and began the practice of his profession, in which he continued until near his death. He was appointed surgeon of the Central Railroad of New Jersey and was nominated for mayor of Elizabeth in 1884. He was a republican in politics and sustained defeat with his party in that year. He was buried with military honors, conducted by Kilpatrick Post, G. A. R., of which he was a member. His wife died some years ago and he left no living near relatives.

